# Telephone follow-up of oncology patients: the contribution of the nurse specialist for a Service-Dominant Logic in hospital

**DOI:** 10.1186/s12913-021-06552-8

**Published:** 2021-06-16

**Authors:** Corinne Rochette, Anne Sophie Michallet, Stéphanie Malartre-Sapienza, Sophie Rodier

**Affiliations:** 1 CleRMa, University of Clermont Auvergne, Clermont-Ferrand, France; 2 Cancer Control Unit, Léon Bérard, Lyon, France

**Keywords:** Service-Dominant Logic, Qualitative study, Telephone follow-up, Healthcare pathway, Patient empowerment, Patient experience, Nurse specialist, Haematology, France

## Abstract

**Background:**

The French healthcare system is characterised by a shift towards outpatient care and the desire to develop telemedicine affirmed in the collective commitment “Ma santé 2022” presented by President Macron in 2018. In France, remote patient follow up has recently been developed in the active phase of cancer treatment inspired by the patient navigation approach used in other countries. According to Service-Dominant Logic (S-D L), patients become more active. Their role in co-production of services is strengthened and their behaviours changed. Telephone follow-ups can contribute to modifying the relationship between the patient and the nurse navigators in charge of it, moving logically from a passive attitude from the patient to a more active one.

**Methods:**

This study was carried out at Léon Bérard, a cancer control unit, in France. It concerned patients treated in an oncohaematology department, who benefited from telephone follow-ups carried out by nurse specialists during the active phase of their treatment. The multidisciplinary research team including social science researchers, physicians and carers developed a research protocol to study this pilot case. Essentially based on a qualitative approach, it was validated by the centre’s management to study this follow-up on patients’ behaviours. The 1st phase of the research, based on 24 semi-structured interviews with patients undergoing treatment undertaken from November 2018 to September 2019, is presented.

**Results:**

The Telephone follow-up was a positive experience for all patients. The action of the nurse specialist helped to develop certain dimensions of in-role and extra-role behaviour that created value. The patients’ discourse has reported a positive follow-up in its clinical dimensions, its psychological dimensions and an enhanced quality of life. We detected a patient activation through their roles but it remained limited. The telephone follow-up also created a patient dependency.

**Conclusions:**

The telephone follow-up is a relevant tool for patients undergoing treatment and it deserves to be more widely deployed. It brings comfort and creates a relationship based on trust but at the same time it limits the emancipation of the patient, which is a central element of the S-D logic and its empowerment.

**Supplementary Information:**

The online version contains supplementary material available at 10.1186/s12913-021-06552-8.

## Background

Health services face strong pressure due to increasing demand and limited resources, whilst at the same time wishing to improve patient care and support. In particular, hospitals have to control costs [1], innovate and adapt their organization for treating chronic disease [2]. Meeting these challenges requires a rethinking of the dominant logic [3]. The collective commitment “Ma santé 2022” presented by President Macron in 2018 and operationalised on July 26th 2019 by the adoption of the law on the organisation and transformation of the health system integrates these issues. Two axes emerge to draw the contours of a new organisation of the French health system. The first is the “outpatient shift”. The development of outpatient care has been a major focus of hospital reforms for a number of years in many countries [4] but France lags behind many OECD countries. The second is telemedicine, again introduced late in France. It is presented in the 2014–2019 cancer plan as a means of improving the care of cancer patients [5]. In oncology, since the start of the Covid-19 health crisis, the emergence of teleconsultation has accelerated [6] and is increasingly seen as an appropriate response to patient care needs [7].

Telephone follow-up is one of the dimensions of telemedicine next to teleconsultation, telediagnosis and other forms of remote care. This is particularly relevant in the case of chronic illnesses such as diabetes, heart failure, depression and increasingly for cancers. Studies show that the use of the telephone can be a very effective means of ensuring that cancer patients can be followed up without having to travel sometimes over long distances. Thus they don’t need to reorganize their diary to free up the time needed, having to bear the costs (travel, parking, childcare, loss of a day or half-day’s work depending on the distance they have to travel) to go to their consultation centre to have a face-to-face consultation [8]. In the field of oncology, studies show that patient follow-up meets several objectives: prevention of complications, collection of medical data for clinical research [9], reduction of costs in terms of public health and early detection of relapses [10]. Although monitoring is costly in terms of medical resources, it is also an indicator of patient-centred cancer services [11]. The follow-up strategies deployed by cancer centres focus essentially on routine follow-ups with clinical examination from oncologists or general practitioners, some had adopted telephone follow-up done by physicians and more often by nurse specialists or nurse navigators. Studies on types of follow-up (follow-up by the general practitioner, patient-initiated or nurse-led follow-up or contact by telephone) have not shown any great difference in terms of the perception of the service provided for the patient [12–16]. However, the ideal structure, method and timing of telephone follow-up as well as the skills required to carry out such care over the phone are often not considered and have not been articulated [8] even if the option of telephone follow-up could dramatically improve the care experience for some patients [8, 17]. Telephone follow-up requires another form of patient participation because of the physical distance. It also requires an adaptation of the health care professionals to this remote relationship because a technological tool is interposed between the patient and the health care professionals; the telephone. The absence of face-to-face meetings changes the way the service is delivered and leads to reintroducing the patient as an active participant in tele-monitoring and transforming the role of patients [18]. The role of patient as a healthcare service co-creator is enhanced. This new organisational design is a manifestation of the S-D L approach [19–21]. This approach rejects the traditional proposition that the service provider exclusively creates value. The *‘logic of interactivity’* involves participating actors co-creating value through an exchange of operant resources such as skills, knowledge and competencies. S-D Logic and co-creation are naturally linked. This logic considers the value as the result of interactions between service providers and beneficiaries [22–25]. Co-production is not an “add on” to services but a core feature of them [26]. S-D L is based on the principle of contributing resources that are integrated by other actors than the main provider-to-provider service [19]. From this perspective, patients can have a role as a co-creator of value and thus participate in the construction of a service in which they are stakeholders, like physicians and healthcare providers [27]. The SD-L proposes a model in which micro-skills, know-how and expertise (operant resources) are mobilised in order to act on tangible resources (operand resources) such as products and material resources [24]. Patients can influence the organisational structure and the nature of the resources used to support them through their in-role behaviours and their extra-role behaviours [28]. The in-role behaviour or customer participation behaviour is a necessary and indispensable behaviour for value creation. It is made up of four sub-dimensions; the search for information, sharing information, responsible behaviour and personal interaction [28]. The extra-role behaviour or customer citizenship behaviour is a voluntary behaviour that generates “extraordinary value” for the organisation but it is not required for successful co-creation of service. It is also composed of four dimensions; feedback, advocacy, help for other patients and tolerance [28]. Behaviours depend on the nature and quality of interactions with staff and on the patient’s situation. The quality of interaction affects not only the satisfaction of the service user (patient) [27] but also their service outcomes [29, 30]. In oncology, there is a lack of research on patient engagement at micro-level encounters [31] and on patient activation [32]. For the founders of S-D L there is a challenge “to understand how wellbeing of individuals is both contingent on and contributes to a dynamic network, in which the resources of the actors are being continually updated” [33]. Regarding the healthcare system and the remote follow-up, it means to study how its evolution contributes to a better integration of the specific resources that are the behaviours of the patient. Changes in terminology are representative of a new way of looking at the patient. The concepts of patient empowerment [34–36], patient partner, patient expert [37, 38], patient engagement [39], patient centred care [40, 41] and therapeutic patient education are all part of this perspective, although SD-L is not always mentioned in these studies. They show a willingness to put the patient back at the core of processes to improve care by adopting approaches based on the patient’s perspectives and experience [42–44] and in a more open approach it signals the growing importance given to the patient’s voice. Patients become true partners who are able to provide avenues for improving the health service and care provided. Their voice contributes to place them in the context of their life pathway and not only in their care pathway. More and more, the patient is considered as the holder of special skills, as an expert of their disease [45, 46]. The patient’s experience becomes a resource on which to draw to create more value [47]. The patient could be considered as a health co-provider [32, 45]. Berry and Seltman [48] described the approaches that represent the application of S-D L to cancer care. For them “*oncology practice provides treatment, but that is a fraction of the patient needs*”.^1^

In the context of the development of S-D L in hospital [32, 49] and remote patient monitoring, the role of the patient is being questioned. Works have focused on the clinical benefits of these monitoring systems but had little documented the role of the patient.

Research has been undertaken on the effect of remote follow-up for pathologies such as diabetes and heart failure, but the field of telephone follow-up in oncology remains poorly documented because it is more recent. According to the Unicancer federation – the federation that regroups cancer centres in France - very few cancer control units have implemented a systematic telephone follow-up during the active phase of treatment. Unicancer encourages its wider diffusion, sharing experiences and results of the centres that have trialled it. The aim of this paper is to report how the telephone follow up (by outgoing phone calls) implemented in an oncology department during the active phase of treatment transforms the behaviours of patients.

## Methods

We focused our approach on the patient perspective using a qualitative approach based on semi-structured interviews. Our methodology allows us to go beyond quantitative approaches based on questionnaires to gain a more detailed understanding of patient behaviour and of the patient’s contribution to the co-creation of health service provision. The contributions are two-fold. Firstly, they provide a reading of patient behaviour in an extended approach of the patient roles in the context of telephone follow-up evaluating their activation. Secondly, it contributes to identifying the critical points of this type of organisational model.

Telephone follow-up of cancer patients called Outpatient Medical Assistance (OMA) was set up at the haematology service of the Léon Bérard Cancer Control Unit (CCU) (Lyon, France) in 2016 as a pilot case [50–52] for this hospital. The purpose of the OMA program is to reduce patient visits to hospital and to develop a real partnership between patients and the hospital via nurse specialists. Our research was designed to study how, in the OMA program, the patient takes part in co-creating the follow-up service. After consulting secondary sources of information, a three-step research protocol^2^ was designed by a research team including social science researchers and medical staff of the hospital. Consent for the implementation of the research was obtained from the hospital’s research department. In this article we present the implementation and results of the first step.

### The case study

The OMA program is part of the medical-scientific project of the haematology department of the Léon Bérard CCU. The OMA program is aimed at patients suffering from haematological malignancies in the active phase of their treatment (intravenous chemotherapy) but also at patients undergoing oral therapy. The hospital’s medical team launched a call for research projects to evaluate this remote follow-up system used for patients treated for cancer. Our academic team, external to the service, applied proposing the research design selected. The OMA program reduces secondary hospitalisations by 50% and improves treatment compliance. The aim of this programme is to improve the patient follow up in the active phase of their treatment: anticipating and managing the risk at home; managing and detecting as early as possible the toxicities that may be caused (fever, mucites, digestive problems, asthenia, pain, neutropenia, anaemia, etc.) by chemotherapy; ensuring proper compliance with treatment; breaking the isolation and rationalising care. This mission is carried out at the Léon Bérard Centre by 3 experienced nurses in haematology. To date, 350 patients have benefited from coordinated follow-up (activity spread over the 3 nurses specialists), of which 32 patients are Young Adult Adolescents (YAA) as part of the development of a partnership with the paediatrics department since June 2017. The current active file is 70 patients/day with the inclusion of around 10 new patients per month.

In this centre, the complexity of care, the hyper-specialisation of units, the multiplication of call lines contributes to considering the unscheduled incoming phone call as an imperfect organisational modality. In this context, an alternative model based on the outgoing phone call (directed from the care unit to the patient) from a resource person (a nurse specialist known as an OMA nurse) has been set up for the time of treatment. During the treatment period, the patient receives two weekly scheduled calls in which the nurse specialist answers the patient’s questions and also assesses the evolution of the patient’s physical and psychological situation. The nurse specialist works closely with the haematologist who follows the patient in order to adapt actions to the evolution of the patient’s state of health. When the patient has finished treatment, the telephone follow-up with programmed calls stops.

### Data collection

We have selected a sample of patients from those included in the OMA telephone follow-ups according to a list of criteria in order to have representation of all categories of patient. The criteria were: women and men, a representation of the various age groups, their geographical location (patients living near the Centre and others geographically more distant), patients living alone and others living in a couple. After presenting the research project to the 25 selected patients, we obtained the voluntary consent to participate from 24 of them.^3^

The two researchers in human and social sciences conducted the semi-structured interviews from November 2018 to September 2019 with patients^4^ from 22 to 94 years old included in the telephone follow-up process (Table 1). The interview guide used was developed for this study (available in supplementary file). Before the interviews, the nurse navigator, who assists the researchers in their contact with patients, presented the medical and social situations of each of them to adapt, if necessary, the physical conduct of the interviews.

**Table 1:** description of the sample of patients interviewed

Patients	Start of medical treatment (month and year)	Age range (years old)	Geographical location distance from the CCU	Marital status	Duration of the interview in minutes	Date of interview
Patient 1	April 2016	61–80 yo	20–25 min	widow	29	06-nov.
Patient 2	August 2018	26–45 yo	10 min	married	20	06-nov.
Patient 3	April 2018	26–45 yo	10 min	single	39	06-nov.
Patient 4	July.2018	26–45 yo	40 min	Co-habiting	21	06-nov.
Patient 5	December 2017	26–45 yo	10 min	married	51	20-nov.
Patient 6	January 2018	26–45 yo	10–15 min	single	34	20-nov.
Patient 7	March 2018	61–80 yo	20–25 min	married	25	20-nov.
Patient 8	December 2017	46–60 yo	30 min (rural area)	married	22	21-nov.
Patient 9	April 2016	26–45 yo	> 2 h road (150 kms)	single	25	21-nov.
Patient 10	April 2018	61–80 yo	20 min	married	16	21-nov.
Patient 11	May 2017	81–100 yo	20–25 min	married	38	21-nov.
Patient 12	July 2018	81–100 yo	10 min	married	18	21-nov.
Patiente 13	October 2017	81–100 yo	10–15 min	widow	23	21-nov.
Patient 14	March 2018	46–60 yo	30–35 min (rural area)	married	41	27-nov.
Patient 15	February 2018	46–60 yo	1 h	divorced	22	27-nov.
Patient 16	September-2018	46–60 yo	35–40 min	married	27	27-nov.
Patient 17	August 2018	61–80 yo	25–30 min	married	30	30-nov.
Patient 18	April 2018	46–60 yo	25–30 min	divorced	12	30-nov.
Parient19	September 2018	18–25 yo	10–15 min	Co-habiting	34	30-nov.
Patient 20	Tune 2018	26–45 yo	1 h28 mn	married	40	11-july
Patient 21	June 2018	46–60 yo	1 h59 mn	Co-habiting	35	11-july
Patient 22	February 2019	46–60 yo	2h08mn	divorced	23	12-sept
Patient 23	March 2019	61–80 yo	22 min	divorced	94	12-sept
Patient 24	January 2019	18–25 yo	8 min	married	44	13-sept

Interviews were structured around four topics: 1° what outgoing telephone monitoring by programmed telephone calls represents for the patients, 2° the interactions (type) with the specialist nurse, 3° the impact of this follow-up on their behaviour and their life, 4° their wishes in terms of the evolution of the system. We recorded and transcribed the 763 min of interviews to carry out a content analysis. We obtained data saturation at the end of the sixteenth interview, but we completed the 24 interviews scheduled.

### Data analysis

After each interview the two researchers listened to the recording before transcription to carry out independently the iterative reading process and thematic and lexical identification. This resulted in an initial classification of verbatims and they discussed the assignment of words and ideas to the key units of analysis using the codification process recommended by Freeman [53]. Once all the interviews had been transcribed, a detailed thematic and lexical content analysis was carried out on all the verbatims using the predefined and adjusted grid [51, 54]. The coding of the data^5^ followed the dimensions of the two types of behaviours (in-role behaviour or customer participation behaviour and extra-role behaviour or customer citizenship behaviour) [28].

## Results

Our results provides a better understanding of the nature of the interaction between patients and nurse specialists from the patients’ perspective. The nurse’s action, because of the important trust created with them (1), helps to develop the in-role behaviour of patients in particular on the sharing of information, but this remains limited on the other dimensions (2). Concerning the patients’ extra-role behaviour, there are existing signs showing its contribution to service co-production, even if it remains poor (3).

### A prerequisite to telephone follow-up: trust between patient and nurse specialists for patient activation

Telephone follow-up transformed the relationship between the patient and the nurse specialist and, more generally, between the patient and the hospital. This relationship is characterised by a strong confidence from the patient in the nurse and his or her skills which facilitate interaction. One of the cornerstones of trust is a physical meeting during the patient’s pathway. Patients need to humanise the remote monitoring service by giving a face to a voice, that is to say the specialist nurse who telephones them twice a week. All of them considered that “*it is important to meet from time to time to put a name to a face and create a relationship of trust”* (I2, I6, I7, I10, I11, I16, I18). But at the same time for all respondents, the content of remote exchanges is identical to a face-to-face discussion, or it would be. Remote monitoring fully satisfies them because it brings comfort and “*It is more practical”* (I4). The smooth running of the follow-up requires a physical meeting at the time of his or her inclusion in the follow-up system. “E*. (i.e. OMA nurse), before I talked to her on the phone, I met her and that is why I was able to have that relationship by phone and put my trust in her”.* (I20).

In the patients’ discourse, there is an important valorisation of the role of the nurse through the roles attributed by the patients to the OMA nurse specialist:

- They are a double-level translator. (1) They make medical information understandable and accessible *“Exchanges make it possible to understand blood tests and side effects”* (I2), *“the nurse is close to us, she interprets and translates the information given by the physician, she takes the time that the physician does not have”* (I3). (2) They translate the patient’s words and ills in order to assess their condition and anticipate certain effects of the treatment and disease.
- They represent a psycho-social support: their role goes far beyond medical follow-up. The patient approach they develop is holistic. “*The OMA nurse also made it possible to manage the family’s constraints. The impact on my life was enormous, it was a huge comfort. She was the only one who could get me back on track” (*I5). *“She is a pillar*” (I6). *“There is humanity settling in. We get used to these comfort calls, we are not alone in the face of illness”* (I17). Patient activation is also a question of the specialist nurse’s involvement and availability. They were, for the patient, a coach who puts them into action in their ability to manage not only the psychological dimension but also the material aspects of everyday life such as family logistics or professional life.
- For some tasks, with a medical content, nurses have a coordinating and substitute role. They made the connection with the physician. *“It was amazing. I didn’t need to ask the physician because I found everything I needed at Léon Bérard Centre.”* (I15)

In case of doubt or need, the first reflex of patients was to contact the OMA nurse. *“If it hadn’t been for this follow-up process, I would have sometimes gone to the emergency room”* (I14). Through this telephone follow-up, the nurses freed the patients from a number of concerns related to their care pathway, allowing them to concentrate on other roles. The scope of activation was not exclusively clinical (compliance with treatment, self-management of adverse effects, etc.).

Thus, the participation of the patient in the service process (in-role and extra-role) in the outpatient telephone follow-up requires considering accurately the central place of the nurse specialist.

### In-role behaviour

A remote patient follow-up means an increased patient participation in the service process due to geographical distance. The patient has to handle a part of the actions, such as identification of symptoms and medication compliance, traditionally done by healthcare professionals when the patient stays in hospital.

#### Search for information

The understanding of the telephone follow-up program was built up along the way when patients become more experienced with it. Moreover, some of the patients did not initially have any particular expectations of the program. They did not know it and/or a small part of them did not understand what it consisted of during the disease announcing consultation. The understanding of the OMA follow up program was not homogeneous. A (minority) part of the interviewees did not understand what the system first consisted of, despite its presentation at the time of the disease announcing. They discovered it along the way. For some patients, it was linked to the amazement caused by the disease announcing. Many of them at this stage of the care pathway were unable to hear nor to listen. *“The physician had told me about it, then Mrs “A” (i.e. the OMA nurse) had been introduced to me, but I discovered the principle of telephone follow-up as I went along”. (I18).*

Very few patients said they seek information on their disease on their own. Their main sources of information were nurses and practitioners; this indicates a strong level of confidence in the health care providers of the CCU.

#### Sharing information

Patients eagerly awaited telephone appointments; some of them prepared for them carefully by writing down the questions they wished to ask. Some even kept a notebook in which they note the effects of the treatment to provide the nurse with the best possible information on their state of health.

Respondents felt perfectly comfortable formulating their feelings at a distance, describing the manifestations of the disease and the effects of the treatment. In this respect, we can say that the interviewed patients, regardless of their age and socio-professional background, were active and had good health literacy (except for the oldest patient who benefited from his wife’s help on this point). It is clear that regular exchanges with the nurse specialist allow for a better, knowledge of the disease and to build very strong trust that makes it possible to “*address very personal or even intimate issues that are not discussed with the physician”* (I5). The trust established between the patient and the nurse specialist facilitates exchange of information. Patients very frequently used the word ‘trust’ when talking about relations with the nurse specialist. Patients have a high level of trust in the nurse specialist. Confidence depends on the skills demonstrated by nurses. For several patients and with regard to their pathology and treatment, the nurse specialist has even higher skills than general practitioners do. In certain situations related to the disease where the general practitioner would be the natural contact, patients exchanged information more easily with nurses.

“*When I had side effects I would first call Léon Bérard (i.e. CCU), I was told, “See your GP”. But in my opinion, only Léon Bérard really has the expertise for this pathology. I have the impression that the general practitioner has a lack of knowledge about the disease; I didn't trust him, so I contact Léon Bérard exclusively”*. (I19)

To be able to share information, patients must feel comfortable addressing very personal and sometimes very intimate concerns that they did not dare to discuss with the oncologist. *“I never felt that there could be a discomfort with the nurse specialist. Rather, she made me feel comfortable because she was so simple; I had no taboos about what I wanted to talk about*.” (I20). Because the same nurse always did the telephone follow-up, she knows the patient very well. She was able to “feel” when he or she was not fine; *“she is able to get out of me things that I wasn’t able to get out to my loved ones because I was afraid of hurting them even more than I do because of my disease. She helped me both physically as well as psychologically.”*(I21).

This role of the patient of information sharing was also largely triggered by the nurse’s attitude in charge of their telephone follow-up. Knowing that it was easy to have information and to share the information you had, thanks to a telephone number behind which there was a benevolent person supports the in-role behaviour of the patient of sharing information. “*This phone number that you can call in any circumstance is worth gold”* (I8). “*I have understood one thing, at least in Léon Bérard, there is a lot of consultation between all persons involved in the pathway. Information circulates. They share information. There is really a great coordination*.” (I23).

#### Responsible behaviour compliance

All the patients questioned were careful to observe scrupulously their treatment. Sometimes respecting the treatment could be a source of anxiety. Some people were afraid of making a mistake because the number of tablets to be taken was large, “*at the beginning I said to myself ‘I’m never going to be able to remember all this*’”. (I23).

The adherence to the observance of treatment was observed on a completely different dimension, which was the observance of the operating hours of the tele-monitoring service. Despite the fact that their condition might require them to act, patients did not take the initiative for action during periods not covered (evenings and weekends) by the outgoing telephone follow-up system. This illustrates the patient’s difficulty with self-management and a low aptitude for (patient) navigation.

#### Personal interaction

On this dimension, we can identify a paradox. Patients had the means to get in touch with competent personnel very quickly for a rapid adjustment of their medical management and their care (personal interaction). However, many said that they wait (1, 2 or 3 days) for the scheduled phone call of the nurse specialist to talk about the symptoms and adverse effects, sometimes very pronounced, that they were experiencing.

“*During the first chemotherapy treatment, I had warts and pimples in my mouth and throat, stomach pains. I told myself that after chemo it's normal, it will disappear. I was in so much pain that I couldn't eat, just a bun soaked in milk. When the nurse called me and asked me if I had eaten, I told her I hadn't. I said no. I couldn’t. It was as if everything was burnt, my mouth, my throat.... She told me “you shouldn't have remained like that for several days of suffering, it's absolutely necessary to call me”.*” (I5)

There was also a high level of respect for the programme and the work done by the staff. Some patients sometimes tried to manage alone so as not to overload the OMA. “*Out of respect, I didn’t mean to disturb them. I’d prepared myself to manage”* (I16). This sentence is also an illustration of the tolerance (a dimension of the extra-role behaviour) as we will see below.

Patients could initiate the telephone call when they had a worrying feeling or a manifestation of the disease or treatment that was particularly pronounced compared to the usual feelings; “*sometimes I call to be reassured*… *I would call before she called me when I was anxious or because I felt a particularly strong symptom... afterwards because the side effects were always relatively the same, after a time I also knew how to manage my condition*” (I19). Over time, both younger and older patients developed a degree of autonomy (but it’s relative), although it was less frequent for older patients.

### Extra-role behaviour

#### Advocacy

All patients interviewed had a very positive perception of the telephone follow-up by the nurse specialist. It was characterised by a high quality of interaction. The skills of nurses were very widely recognised; they contributed greatly to creating a positive experience that patients shared around them with other patients, with their family, friends, or other health professionals (family medicine, community nurses) involved in their healthcare journey.

Patients associated the program with the Léon Bérard CCU before associating it with the service. The OMA telephone follow-up was associated with the expertise of the staff of the Centre. It is an important differentiating factor, as regards the content of patients’ comments, especially for those who had exchanged with patients from other hospitals. They became aware that this type of programme was not widely distributed.

The OMA telephone follow-up program makes a significant contribution to the brand image of the Centre, and this, despite the fact that it concerns only one service and a limited scope of inclusion. *“Léon Bérard is a fabulous machine.”* (I16).

They were very surprised that the follow-up was so intensive in terms of call frequency. They used very strong words to describe it with a strong emotional dimension. “*I didn’t think it could exist and now I don’t understand that it doesn’t exist everywhere”, “It was awesome for me”* (I14).

They promote the programme outside the walls of the hospital.

#### Tolerance and “help for other patients”

The extra-role behaviour close to tolerance is the benevolence and altruism expressed by many patients. Most of them expressed benevolence in the willingness of trying not to overburden nurses in order to spare them and to free up the precious resource they represented for other patients who needed it. There was therefore a desire not to over utilise nurses in order that the greatest number of patients could benefit from their services. Patients clearly expressed an altruistic dimension in their discourse towards nurses and other patient beneficiaries of the program of telephone follow-up. *“They have so much work, and there are so many patients who may need it more than I do*” (I17). In discourses, we can identify expressions that refer to a form of embarrassment for patients who benefit from the telephone follow-up programme compared with other patients treated for cancers who do not have such a service and support. It was for this reason that most of them agreed to share their experience in interview and to contribute to spreading an understanding of the comfort that such a follow-up brings at a critical period of their lives. “*I don’t understand why this service doesn’t exist for all patients*” (I15).

Three of them spontaneously expressed the desire to share their experience of the disease. A young patient said that maybe later she would like to share her experience of the disease and treatment. Another might consider becoming an expert patient. “*I would like to give young people like me with a haematological disease the opportunity to talk to someone who has had this disease.*” (I24). Most of those who accepted our interview were aware that they were taking part in the adjustment of the design of the service and its widest implementation.

#### Feedback

Feedback was not directly expressed in the discourse of patients. For the patients, having accepted to participate in our study constitutes a manifestation of extra-role behaviour*. “I am happy to be able to speak with you today and answer your questions because the nurse has been so great with me for months and months, so helpful and available that I would really like to highlight what she has done for me,” ... “to testify and allow other people to benefit from it.”* (I20).

The dimension of the extra-role behaviour of the patient is also a dimension of the OMA follow-up.

## Discussion

This article highlights the perception that patients of an oncology department had of the telephone follow-up (tele-monitoring) set up in the active phase of their treatment and its influence on their in-role and extra-role behaviours. This research confirms that the value created is the result of interactions between service providers and beneficiaries [19, 20, 23, 24]. Three findings come to light.

Firstly, our results showed that interactions between service providers and beneficiaries were largely determined by nurse specialist attitudes, illustrating a low patient leadership and an interaction concentrated on this duo. The S-D L [23, 33] was expressed in a weak, incomplete and closed form. Analysis of patient discourse showed that the large majority of interactions focus on the nurse-specialist and patient duo. The main marker of this relationship is a highly developed trust between the patient and the nurse confirming (for France) the results of the Dec. 1–172,020 Gallup poll that: Nurses: The most trusted professional.^6^ In the case of research on remote telephone follow-up, it was natural for patients to focus on their relationship with the nurse, but patients reported low levels of interaction with the oncologist, the GP and other patients. Yet patients were indeed a partner [37, 38]. Without their extended feedback on how they experience the treatment and the illness, the provision of care services could not be adapted in a more integrated approach. In their speech, the words used showed that they didn’t see themselves in this way. Our study showed different levels of activation depending on the in-roles and extra-roles of patients for a more detailed approach to this issue.

In-role behaviour focused on the dimensions of sharing information and personal interactions. Sharing information was stimulated by the nurse’s facilitating attitude and the strong confidence she inspired in the patient. The nurse sought to assess the patients’ physical and mental state of health in their bi-weekly exchanges so that the medical and nursing team could provide the best response in terms of adjusting treatment and care (adjusting appointments, anticipating possible hospitalisation during times when the remote monitoring service was not active). For personal interaction, if present, our results showed that patients limited themselves by not wanting to take the initiative of contact (in some cases) with the nurse for fear of overloading her even if their situation deteriorated. These elements show the need to change the patients’ attitude in order for them to be real actors in their own health and in the care process and thus to empower the patient [34–36] and enhance patient-centred care [40, 41]. The patient centred care approach that, in previous research, has focused on the evolution of practices and organisational culture of service providers (hospitals) must also explore more strongly the side of service beneficiaries (patients) [27]. This is more than just therapeutic education, it must be part of the patient navigation approach that, among other things, is intended to increase the patient’s autonomy [53] to develop a healthcare self-management culture.

Secondly, patients cannot be considered as a complete resource integrator [19] from the perspective of the integrated care approach developed by authorities and academic research. Patient leadership is poorly developed. This leads the nurses to maintain a mothering attitude. This is due to a relationship that the patient perceives as unbalanced. The passive dimension of the behaviour is dominant. The words and expressions used make little reference to an active and engaging dimension of the patient [39].

Our research highlighted the difficulty of making patients more active in the health care system, even within the framework of remote follow-up, where patients could have a more active attitude. They can provide operant resources (expertise in the disease and treatment experience) but they do not provide them spontaneously. If we can observe an in-role and extra-role evolution [28], it is still insufficiently asserted for patients to gain management autonomy and develop true leadership on their navigation of health services. It is up to the staff with whom they are in contact to guide them in providing these resources, even if patients do not perceive themselves as directly active, they nonetheless create value. An explanation could be found in a more patient-centred approach to the telephone follow-up than on a person centred approach [41] but also in a weak level of health literacy which is a general characteristic of French people [55]. Because of a low level of health literacy people could not know how to manage their healthcare by themselves and how to navigate the health system. As our results showed, from this point of view, nurses contribute to patient education, by accompanying them towards self-management of certain aspects related to their illness [53]. The approach remains more clinical than holistic. It is clear from our results that the role of resource integrator [19] is played by the nurse and not by the patient [53, 55].

Thirdly, our results showed that the implementation of remote patient monitoring services far from making patients more autonomous creates a dependency. Thus, patients fear the post-treatment phase when the telephone follow-up stops. Some of them felt lost. This illustrates how difficult it is for the patient to break free from the codes of care behaviour enshrined in the history of our healthcare system. The extra-role behaviour of patients, which refers to the voluntary behaviour to create extraordinary value, can be identified above all in the advocacy dimension, even if the other dimensions (tolerance, help for patient and feeback) are present, they are less noticeable and pronounced [28]. The Léon Bérard centre’s outpatient telephone follow-up programme makes a strong contribution to the centre’s brand image and therefore creates intangible value (good image and reputation). It is a strong differentiating factor.

Finally, our study also contributes to work on follow-up systems [14, 15]. It confirmed that a well-designed nurse-led follow-up can potentially enhance the co-creation value of the health service system by facilitating a better response to the medical and psycho-social needs of individual patients [15] and can also support more autonomous patient behaviours. This, however, requires an evolution from a patient-centred approach to a person-centred approach that is less motherly. It is the pillar of a stronger affirmation of S-D L in health services based on patient empowerment to give more influence to their roles.

### Contributions

Our work makes three types of contributions; a contribution to the literature on the S-D L and integrated care pathways, a methodological contribution and managerial contributions.

Our study showed the implication of the concept of S-D L within a setting specific to oncology patients of telephone follow-up using the in-role and extra-role approach. It contributes to the S-D L literature transposed to care services management [27, 32] and to the integrated care pathway literature [56] by showing how much we have to rethink the place of the patient in health services by relying on the nursing staff. Our work showed the paradoxical effects of setting up a remote monitoring service between the development of patients’ roles and an autonomy which remains relative (in-role and extra-role) and the creation of dependence on the monitoring service offered.

The second contribution concerns the methodology used. The study of the pilot case [51] was based on a qualitative approach [54] centred on patients’ discourse to study their behaviour in the context of telephone monitoring. Even if it is not without limits, the qualitative approach allowed for a more refined view of the issues. Our approach responded to the demand from health authorities and health researchers to develop rigorous qualitative studies around stakeholder behaviour to shed new light on the issues raised by the health system and health services. This is particularly evident in France through the development of ancillary studies in the social sciences in medical research programmes, as our research illustrates. We had taken care to develop a rigorous research design that has been systematically subjected to evaluation and discussion. In qualitative research, the quality of the contribution is based on the ability to provide proof of the robustness of the research design (tracing of data collection and analysis) without leaving aside the interpretation of the data by the researchers [54], which is a characteristic and richness of this type of research.

Our third contribution concerns managerial recommendations and are formulated according to two types of value creation behaviour [28].The outpatient telephone follow-up only operates during the day and excludes weekends. So, for the patient, the interruption to the service outside these times could have been a significant problem. Even if most patients said they can wait until Monday, sometimes despite significant inconvenience, the development of a customer-centric organizational culture requires thinking about real service solutions for these times (evenings and weekends). In these times, the patient initiated the contact by calling the emergency service (the only telephone number to call in the evenings and at weekends) which takes over, often with resident doctors. It is important to offer a service that is a continuation to that offered during the week, such as an incoming call number to a specialist nurse. This could further strengthen the patient’s role behaviour by promoting information sharing and rapid reaction of the patient if a symptom occurs without having to wait for the day of the scheduled call. It is also necessary to communicate to show patients how they can act and be more active in the treatment and follow-up process. These elements can be used to enhance patient navigation [53]. For example, during the interviews, patients did not mention their participation in the workshops set up at the centre. It is necessary to find another formula so patients can benefit from it since this can help to develop their health literacy. Due to the remote monitoring, they benefit from less travel to the centre than do other patients. It would therefore be relevant to offer remote information materials such as a video presenting the OMA programme and how it works (many didn’t understand it when first presented), and putting patients who wish to share their experience of the disease in contact with each other to organise the conditions for their extra-role behaviour to be expressed.

The follow-up process concerns the active phase of treatment. For some patients, the cessation of follow-up at the end of treatment was experienced as abrupt even if they had the telephone number of the nurse who followed them during the active phase of treatment: the official follow-up stopped. Some people regretted that the transition to post-treatment was not thought out in organizational terms by proposing a smooth transition. For some patients, there was a strong dependence on the remote follow-up. We recommend proposing a lighter follow-up solution during the first months after treatment in order to allow the patient to disconnect gradually from the CCU. This could take the shape of a call every week in the 1st month, then one every 15 days in the 2nd month and finally one call per month until the 6th month.

Our research emphasizes the key role of the nurse specialist with recognized skills and the difficulty in helping the patient to be more autonomous and an actor in the health service. The training of nurses must also give special attention to the issue of patient empowerment [36, 38].

### Limitations and future research

This study has various limitations. This is a specific case of a telephone follow-up in a specific setting, the haematological department of Léon Bérard centre concerning patients included in this outpatient remote follow-up. All these specific features require additional research by replicating this research design on another cancer pathology (breast cancer) or department. It would also be interesting to replicate this study on other types of remote monitoring processes to identify if, in some cases, value creation behaviours are higher than in others and show the influence of the type of remote monitoring system on patient behaviours and autonomy. Literature reports very little difference in patient satisfaction according to the different types of remote monitoring (follow-up by the general practitioner, patient-initiated or nurse-led follow-up or contact by telephone) [12–16]. It would be interesting to see if it is the same for the value creation behaviours in terms of the in-role and extra-role dimensions. Some remote monitoring program probably leaves more initiative to the patient. From this point of view, it is essential to study the case of patients followed for a chronic disease in order to identify possible peculiarities in value creation behaviour. Finally, because the service creation process is bi-directional, the behaviour of value creation has to be explored on the side of caregivers [19, 23, 24]. Moreover, our results showed the central role of the nurse specialist. This is the second limitation of our research.

In this paper we have presented only the first step of our research program. This study will be completed by the use of data from the semi-structured interviews with the nurses and the questionnaire sent to all the patients included in the system. By completing this first step we are working to strengthen the external validity of our work.

## Conclusion

This study complements research on patient telephone follow-up through co-creation of value behaviours. We show the patient’s value-creation behaviours based on in-role and extra-role behaviours in the context of a remote outpatient telephone follow-up programme for patients with hematologic cancer. Based on 24 semi-structured interviews of patient beneficiaries of a telephone follow-up, we highlight the facilitating role of the nurse specialist who acts more particularly on the dimensions of in-role behaviour (sharing information, responsible behaviour) but also, in an attenuated form, on extra-role behaviour (advocacy, tolerance). This exploratory study shows the need to guide the patient towards greater autonomy and more active attitudes, in particular on the ‘personal interaction’ and ‘feedback’ dimensions, and to develop nursing training sessions on patient empowerment.

## Supplementary Information

**Additional file 1.** Interview guide with patients concerning the scheduled telephone follow-up done by a nurse specialist during treatment (CCU Léon Bérard).

**Additional file 2.** Principles of the coding approach applied to verbatims.

## Data Availability

Dataset files are available upon request from corresponding author.

## Abbreviations

S-D L: Service Dominant Logic
CCU: Cancer Control Units are hospitals dedicated to the fight against cancer (screening, treatment, follow-up)
OMA: Outpatient medical assistance is the name of the program of telephone follow-up based on calls scheduled and undertaken by nurse specialists

## Footnotes

1 Berry L. Profiles in improvement. Paper presented at: Institute for Healthcare improvement, December 2014

2 The 3 steps were: interviews of patients, interviews of nurse navigators and a questionnaire sent to all the patients included in the telephone follow-up.

3 Informed consent was obtained for each patient in verbal format, format validated by the hospital’s research department.

4 With the medical and care team, we selected 25 patients who were representative of the profiles of the patients treated. After being informed, they agreed to be interviewed. 1 patient could not be interviewed.

5 See the supplementary file for the coding grid used.

6 https://news.gallup.com/poll/328136/ethics-ratings-rise-medical-workers-teachers.aspx

## References

[1] Fuchs VR (2008). Three “inconvenient truths” about health care. N Engl J Med.

[2] Bodenheimer T (2008). Transforming practice. N Engl J Med.

[3] White KR, Thompson S, Griffith JR (2011). Transforming the dominant logic of hospitals. Biennial Rev Healthc Manag.

[4] Planel MP, Varnier F. Les fondements du virage ambulatoire. In Presses de l’EHESP; Hygée Editions. 2017. see for that https://www.presses.ehesp.fr/produit/fondements-virage-ambulatoire/.

[5] Ohannessian R, Guettier C, Lemaire A, Denis F, Ottavy F, Salle FG, Gutierrez M (2017). L’utilisation de la télémédecine pour la prise en charge du cancer en France en 2016. Eur Res Telemed.

[6] Suc L, Daguenet E, Louati S, Gras M, Langrand-Escure J, Sotton S, Magné N (2021). La téléconsultation pour le suivi après radiothérapie du cancer de la prostate: une place à l’innovation numérique en cancérologie. Cancer Radiother.

[7] Knudsen KE, Willman C, Winn R (2021). Optimizing the use of telemedicine in oncology care: postpandemic opportunities. Clin Cancer Res.

[8] Cusack M, Taylor C (2010). A literature review of the potential of telephone follow-up in colorectal cancer. J Clin Nurs.

[9] Brada M (1995). Is there a need to follow-up cancer patients?. Eur J Cancer.

[10] Ferrua M, Minvielle E, Fourcade A, Lalloué B, Sicotte C, Di Palma M, Mir O (2020). How to design a remote patient monitoring system? A French case study. BMC Health Serv Res.

[11] Calman F, Hine D (1995). A policy framework for commissioning cancer services. Expert Advisory Group on Cancer to tke Chief Medical Officiers of England and Wales.

[12] Wasson J, Gaudette C, Whaley F, Sauvigne A, Baribeau P, Welch HG (1992). Telephone care as a substitute for routine clinic follow-up. JAMA.

[13] Gulliford T, Opomu M, Wilson E, Hanham I, Epstein R (1997). Popularity of less frequent follow up for breast cancer in randomised study: initial findings from the hotline study. BMJ.

[14] Beaver K, Latif S, Williamson S, Procter D, Sheridan J, Heath J (2010). An exploratory study of the follow-up care needs of patients treated for colorectal cancer. J Clin Nurs.

[15] Koinberg IL, Fridlund B, Engholm GB, Holmberg L (2004). Nurse-led follow-up on demand or by a physician after breast cancer surgery: a randomised study. Eur J Oncol Nurs.

[16] Kimman ML, Voogd AC, Dirksen CD, Falger P, Hupperets PS, Keymeulen KB (2007). Follow-up after curative treatment for breast cancer: why do we still adhere to frequent outpatient clinic visits?. Eur J Cancer.

[17] Mir O, Ferrua M, Fourcade A, Mathivon D, Duflot-Boukobza A, Dumont SN (2020). Intervention combining Nurse Navigators (NNs) and a mobile application vs. standard of care (SOC) in cancer patients (pts) treated with oral anticancer agents (OAA): results of CAPRI, a single-center, randomized phase 3 trial. ASCO 2020 annual meeting.

[18] Andersen T, Bjørn P, Kensing F, Moll J (2011). (). Designing for collaborative interpretation in telemonitoring: re-introducing patients as diagnostic agents. Int J Med Inform.

[19] Vargo SL, Lusch RF (2004). Evolving to a new dominant logic for marketing. J Mark.

[20] Lusch RF, Vargo SL (2006). Service-dominant logic: reactions, reflections and refinements. Mark Theory.

[21] Lusch RF, Vargo SL, Lusch RF, Vargo SL (2006). Service-dominant logic as a foundation for a general theory. The service-dominant logic of marketing: dialog, debate, and directions.

[22] Emanuel EJ, Emanuel LL (1992). Four models of the physician-patient relationship. JAMA.

[23] Grönroos C (2011). Value co-creation in service logic: a critical analysis. Mark Theory.

[24] Barnes DC, Collier JE, Lueg JE (2009). Reevaluating the theoretical reasoning regarding market-entry position from a service-dominant logic perspective. J Mark Theory Pract.

[25] Ballantyne D, Varey RJ (2006). Creating value-in-use through marketing interaction: the exchange logic of relating, communicating and knowing. Mark Theory.

[26] Osborne SP, Strokosch K. It takes two to tango? Understanding the co-production of public services by integrating the services management and public administration perspectives. Br J Manag. 2013;24. 10.1111/1467-8551.12010.

[27] Rehman M, Dean AM, Pires GD (2012). A research framework for examining customer participation in value co-creation: applying the service dominant logic to the provision of living support services to oncology day-care patients. Int J Behav Healthc Res.

[28] Yi Y, Gong T (2013). Customer value co-creation behavior: scale development and validation. J Bus Res.

[29] Normann R (1991). Service management: strategy and leadership in service business.

[30] Katz SJ, Lantz PM, Janz N, Fagerlin A, Schwartz K, Liu L (2005). Patient involvement in surgery treatment decisions for breast cancer. J Clin Oncol.

[31] Hardyman W, Daunt KL, Kitchener M (2015). Value co-creation through patient engagement in health care: a micro-level approach and research agenda. Public Manag Rev.

[32] Joiner KA, Lusch RF (2016). Evolving to a new service-dominant logic for health care. Innov Entrep Health.

[33] Vargo SL, Lusch RF (2017). Service-dominant logic 2025. Int J Res Mark.

[34] Anderson R, Funnell MM (2010). Patient empowerment: myths and misconceptions. Patient Educ Couns.

[35] Bravo P, Edwards A, Barr PJ, Scholl I, Elwyn G, McAllister M (2015). Conceptualising patient empowerment: a mixed methods study. BMC Health Serv Res.

[36] Aujoulat I, d’Hoore W, Deccache A (2007). Patient empowerment in theory and practice: polysemy or cacophony?. Patient Educ Couns.

[37] Karazivan P, Dumez V, Flora L, Pomey MP, Del Grande C, Ghadiri DP (2015). The patient-as-partner approach in health care: a conceptual framework for a necessary transition. Acad Med.

[38] Pomey M, Lebel P, Clavel N, Morin E, Morin M, Neault C (2018). Development of patient-inclusive teams: toward a structured methodology. Healthc Q.

[39] Barello S, Graffigna G, Vegni E. Patient engagement as an emerging challenge for healthcare services: mapping the literature. Nurs Res Pract. 2012;2012. 10.1155/2012/905934. 10.1155/2012/905934 PMC3504449 23213497

[40] Davis K, Schoenbaum SC, Audet AM (2005). A 2020 vision of patient-centered primary care. J Gen Intern Med.

[41] Håkansson EJ, Holmström IK, Kumlin T, Kaminsky E, Skoglund K, Höglander J (2019). “Same same or different?” a review of reviews of person-centered and patient-centered care. Patient Educ Couns.

[42] Delnoij DM (2009). Measuring patient experiences in Europe: what can we learn from the experiences in the USA and England?. Eur J Pub Health.

[43] Manary MP, Boulding W, Staelin R, Glickman SW (2013). The patient experience and health outcomes. N Engl J Med.

[44] Wolf JA, Niederhauser V, Marshburn D, LaVela SL (2014). Defining patient experience. Patient Exp J.

[45] Batalden M, Batalden P, Margolis P, Seid M, Armstrong G, Opipari-Arrigan (2016). Coproduction of healthcare service. BMJ Qual Saf.

[46] Englander R, Holmboe E, Batalden P, Caron RM, Durham CF, Foster T (2020). Coproducing health professions education: a prerequisite to coproducing health care services?. Acad Med.

[47] Pine BJ, Pine J, Gilmore JH (1999). The experience economy: work is theatre & every business a stage.

[48] Berry LL, Seltman KD. Management lessons from Mayo Clinic: inside one of the world’s most admired service organizations: McGraw-Hill Professional Publishing; 2008. p. 4. ISBN-13: 978-1260011838.

[49] Vargo SL, Akaka MA (2009). Service-dominant logic as a foundation for service science: clarifications. Serv Sci.

[50] Yin RK (1994). Discovering the future of the case study. Method in evaluation research. Eval Pract.

[51] Patton MQ. Qualitative research & evaluation methods: integrating theory and practice. 4th ed: Sage Publications; 2014. p. 832. ISBN-13: 978-1412972123.

[52] Rowley J (2002). Using case studies in research. Manage Res News.

[53] Freeman HP (2006). Patient navigation: a community based strategy to reduce cancer disparities. J Urban Health.

[54] Saldaña J. The coding manual for qualitative researchers: SAGE Publications Limited; 2021.

[55] Sørensen K, Pelikan J, Röthlin F, Ganahl K, Slonska Z, Doyle G (2015). Health literacy in Europe: comparative results of the European health literacy survey (HLS-EU). Eur J Pub Health.

[56] Mériade L, Rochette C (2021). Integrated care pathway for breast cancer: a relational and geographical approach. Soc Sci Med.

