# Supplementary material for: Telephone follow-up of oncology patients: the contribution of the nurse specialist for a Service-Dominant Logic in hospital
Source: BMC Health Serv Res. 2021 Jun 16;21:580. doi: 10.1186/s12913-021-06552-8 (PMC8206872; doi:10.1186/s12913-021-06552-8)
Supplement: Supplementary file 1 — Additional file 1. Interview guide with patients concerning the scheduled telephone follow-up done by a nurse specialist during treatment (CCU Léon Bérard). [file 12913_2021_6552_MOESM1_ESM.docx]

**Supplementary material file on research methodology**

**Telephone follow-up of oncology patients: the contribution of the nurse specialist for a Service-Dominant Logic in hospital**

Corinne Rochette, Anne-Sophie Michallet, Stéphanie Malartre-Sapienza, Sophie Rodier

**Interview guide^[[1]](#footnote-1)^ with patients concerning the scheduled telephone follow-up done by a nurse specialist during treatment (CCU Léon Bérard)**

**1^st^ theme: what does telephone follow-up represents for you?**

*Reminders (if the patient does not spontaneously address certain points).*

**1.1. Place and effects of the follow-up in and on the everyday life**

- How important is this remote contact for you? Why?

- What about your general satisfaction with this follow-up process? Could you detail the different aspects of your satisfaction and dissatisfaction?

- Do you think this phone follow-up is as an equivalent to a face-to-face meeting with the nurse specialist? Why?

- Do you feel that you are well followed? Why?

**1.2. Time dedicated to remote conversation**

- What do you think about the time allocated for these conversations? Estimate: what is the average time allocated for the telephone calls with the nurse? The shortest and longest duration?

- What do you think of the frequency of telephone contacts?

**2^nd^ Theme: could you detail your interactions with the nurse specialist?**

*Reminders (if the patient does not spontaneously address certain points).*

**2.1. Preparation of discussions**

Do you prepare the phone encounters? Before the telephone appointment, do you write down your questions or the things you would like to discuss with her-him?

**2.2. Content of discussions**

- Could you describe interaction with the nurse? Could you tell us more about the content of your discussions?

- What are the most common topics you discuss with the nurse specialist? What do you find as particularly important? The things that you would like to discuss and that are not? Things that you don't dare to discuss?

- What could you tell us about the confidence-level placed in this telephone follow-up? With the nurse specialist? Do you feel comfortable with her?

- Do discussions with nurse specialist increase your understanding of the disease and help you to better understand your health state?

- Do you address all or only some of the questions you have about the illness and treatment? Do you only discuss certain topics with the nurse? Tell us the reasons why? Do you feel that this was not the object of these phone appointments? Do you feel uncomfortable doing it over the phone? Do you have difficulties in finding the good words to express your feelings, your physical and mental state?

- Do you feel that this phone discussion was more spontaneous than a face-to-face meeting?

- What about your satisfaction about the nurse specialist advice and ability to answer your questions?

**3^rd^ Theme: the impact of this follow-up on your everyday life**

*Reminders (if the patient does not spontaneously address certain points).*

- What is the impact of this follow-up on the manner you experience the everyday treatment?

- What do the effect of this phone follow up has on your life (impacts on social, family and professional life)?

- Do you act differently because of the phone follow-up? Do you change yours habits, behaviours and attitudes toward disease?

- Does it offer you comfort? If so, in which way; Anticipation of the effects of the treatment, decrease in anxiety, decrease in travel to see your doctor, oncologist or pharmacist to get information and report on the effects?

- What is your first reaction in case of adverse effects; to contact the nurse dedicated to your follow-up, your GP, emergency services or someone else?

- Do you notice effects of this follow-up in terms of well-being? If yes or no, what are the reasons why?

- Do there were any dimensions of this follow-up that were a source of anxiety or fears?

**4^th^ theme: your wishes in terms of the evolution of telephone follow-up.**

*Reminders (if the patient does not spontaneously address certain points).*

- do you have any particular expectations regarding this stage in your care pathway? Do you have any ideas on how we could support and guide you during this step?

- In your opinion, does this regular remote contact compensate for the geographical distance? Why?

- Do you perceive this remote follow-up as a complement, a substitute for face-to-face follow-up?

- How do you envisage the end of this follow-up at the end of your treatment?

- Are there wishes concerning this service? What could you tell us about the limits of this process? Do you have any points that should be considered to improve this phone follow-up process? To improve your satisfaction?

- Would you like to be more involved in the follow-up process? In which way?

1. Translated from french [↑](#footnote-ref-1)
