# Supplementary material for: Telephone follow-up of oncology patients: the contribution of the nurse specialist for a Service-Dominant Logic in hospital
Source: BMC Health Serv Res. 2021 Jun 16;21:580. doi: 10.1186/s12913-021-06552-8 (PMC8206872; doi:10.1186/s12913-021-06552-8)
Supplement: Supplementary file 2 — Additional file 2. Principles of the coding approach applied to verbatims. [file 12913_2021_6552_MOESM2_ESM.docx]

**Supplementary material file on research methodology**

**Telephone follow-up of oncology patients: the contribution of the nurse specialist for a Service-Dominant Logic in hospital**

Corinne Rochette, Anne-Sophie Michallet, Stéphanie Malartre-Sapienza, Sophie Rodier

**Principles of the coding approach applied to verbatims**

- ***Coding grid of the verbatims referring to the patient behaviours built on the work of Yi and Gong, 2013***

| Types of behaviours | Dimensions | Examples of the patient behaviours |
| --- | --- | --- |
| In-role behaviour or customer participation behaviour | Search for information | To understand how he or she should behave or act |
|  | Sharing information | To help the physician establish the correct diagnosis |
|  | Responsible behaviour | Compliance with drug treatment |
|  | Personal Interaction | React as soon as a symptom or adverse effect occurs |
| Extra-role behaviour or customer citizenship behaviour | Feedback | Feedback provided to doctors and carers on the experience of the disease |
|  | Advocacy | Positive testimonials on care |
|  | Help for other patients | Accompaniment of other patients, expert patients |
|  | Tolerance | Patience and understanding when difficulties arise in carrying out the service or when the service does not meet expectations. |

- **Coding grid of the verbatims referring to perception of the nurse roles view from the patients**

| Perception of the Nurse positioning view from patient | Medical support | Explanation of adverse effects of treatment, Information on bioanalysis |
| --- | --- | --- |
|  | Psychological support | Identification of the patient's psychological fragility, reassurance, adaptation of care |
|  | Logistic support | Organisation of medical examinations and consultations, adaptation of care to the personal constraints of patients, links with physicians, with the city' s medical services. |
